# Emotional Regulation, Coping, and Resilience in Informal Caregivers: A Network Analysis Approach

**DOI:** 10.3390/bs14080709

**Published:** 2024-08-13

**Authors:** Anna Panzeri, Gioia Bottesi, Marta Ghisi, Cecilia Scalavicci, Andrea Spoto, Giulio Vidotto

**Affiliations:** 1 Department of General Psychology, University of Padova, 35131 Padua, Italy; 2 Unità Operativa Complessa (UOC) Hospital Psychology, Padua University Hospital, 35131 Padua, Italy

**Keywords:** caregiving, informal caregivers, family caregivers, coping strategies, emotion regulation, resilience, health psychology, psychometric network analysis

## Abstract

Public health emergencies such as the COVID-19 pandemic can further strain the mental health of informal caregivers who provide unpaid assistance to family members or friends who need support due to illness, disability, or aging. However, there is a lack of research exploring the resources and adaptive strategies that promote resilience in informal caregivers. This cross-sectional study used psychometric network analysis to model the interplay between coping strategies, emotion regulation, trait resilience, and anxiety and depression symptoms in 351 Italian informal caregivers. The results showed that coping through a positive attitude, emotional reappraisal, and trait resilience were the most central and interconnected nodes in the network. These adaptive strategies buffered against the negative impact of anxiety and depression symptoms, providing valuable insights into the mechanisms underlying resilience and well-being in informal caregivers. Clinically, it is crucial to assess and foster these resilience-promoting factors (positive attitude coping, cognitive reappraisal, and trait resilience) to help mitigate the mental health challenges faced by informal caregivers, especially in the context of public health crises such as the COVID-19 pandemic.

## 1. Introduction

Since the last century, dramatic scientific progress has advanced the quality and accessibility of therapies, medical treatments, and assistance worldwide, significantly enhancing health standards in the early twentieth century [1]. Consequently, life expectancy has increased for those born with and those who acquired a medical condition, leading to progressive aging of the population [2]. Advancements in medicine have also resulted in a growing number of people suffering from aging-related, chronic, and often debilitating medical conditions, which limit daily functioning and require intensive and/or long-term assistance [3,4].

In this regard, the concept of Disability-Adjusted Life Years (DALY) [5] is a critical tool in public health for assessing the burden of diseases, particularly chronic non-communicable diseases (e.g., heart disease, diabetes, cancer). It provides a comprehensive view of the impact of health conditions on populations by combining two key components: Years Lived with Disability (YLD) and Years of Life Lost (YLL).

Individuals with medical conditions often require two types of assistance. On the one hand, professional assistance is provided by formal caregivers (e.g., nurses, physiotherapists, physicians, etc.), who are paid professional figures who provide care to their clients or patients. On the other hand, additional help for daily life activities is often necessary and provided by informal caregivers, who are family members or friends providing unpaid care and support to their loved ones with chronic illnesses, disabilities, or age-related needs [3]. Informal caregivers—also known as family caregivers—help care receivers with various activities, e.g., practical, emotional, economical, and social. Practical tasks include medical care and treatment, accompanying them to medical appointments, assisting in activities of daily living (e.g., cooking and cleaning the house), and personal care (e.g., helping with dressing and washing). Informal caregivers also provide emotional support and help make decisions [6,7]. Moreover, informal caregivers often take on these responsibilities without the adequate training, knowledge, skills, and resources to perform the broad tasks associated with this role.

Being an informal caregiver is a challenging task from different perspectives, including the physical (e.g., fatigue and sleep problems), psychological (e.g., fear and uncertainty), social (e.g., family tensions and social isolation), occupational, and spiritual (e.g., difficulty in redefining life purpose and meaning) levels [8]. The role of informal caregivers can be physically, emotionally, and mentally demanding, putting caregivers at risk of burnout, depression, and other negative health outcomes [9,10].

Despite this evidence, the needs of informal caregivers, the concerns that may derive from providing care, and the physical and mental health issues that may arise are often not considered. Indeed, in the literature, informal caregivers are often referred to as the “invisible patients” [11] or “hidden patients” [12], due to the demanding nature of their role, which can have a profound impact on their emotional and physical well-being.

### 1.1. The Pandemic Burden for Informal Caregivers

The diffusion of the COVID-19 pandemic posed an additional burden on informal caregivers’ condition in various ways, both practically and psychologically (for reviews, [13,14,15]). In one review, Bailey et al. [13] identified four main themes from the experiences of informal caregivers during the pandemic.

The first theme is the fear of virus transmission and a general sense of danger. Most informal caregivers reported fear and anxiety about infecting the patient [15]. Moreover, COVID-19 represented a survival threat [16] both for informal caregivers in the first person and for patients—already in precarious health conditions and somehow depending on the informal caregivers’ health—leaving room for anxiety about future health conditions of the dyad [17].

This leads to the second theme, which is uncertainty, not only about present and future health, but due to the limited information regarding the continuously changing rules about restrictions, lockdowns, and new care systems, along with the economic uncertainty caused by widespread work suspensions [18].

The third theme consists of the burden due to protective measures, closures of support services, and heightened expectations on informal caregivers. Indeed, the pandemic required adopting additional hygienic procedures beyond the preexisting ones and resulted in a reduction in or even closure of support services for informal caregivers and patients [19]. Moreover, access to some sanitary treatments and health services was hindered or postponed because medical efforts had to focus on treating acute COVID-19 conditions. Consequently, even more tasks, responsibilities, and expectations were left on informal caregivers, resulting in additional burdens.

The fourth and last theme is social disconnection. Isolation policies and confinement reduced non-caregiving activities and made it more difficult for informal caregivers to maintain contact with their social support network [20,21]. Thus, opportunities for meetings and emotional support were limited, leading to increased feelings of isolation and loneliness [22].

Overall, scientific evidence showed that, during the COVID-19 pandemic, caregiving demands intensified, exacerbating isolation, stress, and high levels of negative emotions, up to experiencing symptoms of anxiety and depression (for reviews, [13,14,15]). Anxiety and depression can be considered as unwanted negative psychological outcomes and can configure a variety of clinical conditions with increasing severity. If sustained for a prolonged time, anxiety can, in turn, lead to depression by shifting from worry to rumination, focusing on the inevitability of worst-case scenarios, and sowing the seeds of hopelessness [23,24].

### 1.2. Caregivers’ Resources: Coping Strategies, Emotional Regulation, Resilience

Despite this dismal scenario, to buffer the negative outcomes of anxiety and depression, informal caregivers can rely on resources such as emotional regulation strategies, coping mechanisms, and trait resilience.

Two main emotional regulation strategies are the most prominent and studied. Cognitive reappraisal consists of thinking in advance about a situation to reappraise its meaning and subsequent emotional effects, both cognitive and behavioral. The suppression of emotional expression consists of reducing or inhibiting only the behavioral expression of emotions and feelings after they have been generated and experienced, leaving untouched the cognitive experience [25]. Evidence shows that cognitive reappraisal has more favorable outcomes than suppression of emotional expression in terms of affective responding, well-being, and social functioning [26].

To deal with illness-related issues, informal caregivers may use coping strategies, defined as the set of cognitive and/or behavioral efforts undertaken by people to manage external or internal situations assessed as exceeding their resources [27]. Coping strategies may be associated with or mediate psychological experiences related to caregiving [28,29,30]. A variety of coping strategies exist to deal with difficult situations and emotional states, and the most relevant are: adopting a positive attitude toward the problem with acceptance and a resilient attitude; focusing on the problem with cognitive and practical efforts to solve it; relying on social support from peers like friends and relatives who can provide advice and comfort; avoiding and negating the problematic/aversive stimuli; and relying on transcendental/religious coping to seek comfort from God by praying [31,32].

Furthermore, trait resilience is the ability to adapt and cope effectively in the face of adversity, trauma, tragedy, threats, or significant sources of stress [33]. It is an important concept in clinical psychology, as it can help to explain why some individuals can maintain their well-being and functioning despite experiencing significant challenges or stressful life events [34]. For informal caregivers, trait resilience is particularly crucial as individuals with higher levels of trait resilience are better equipped to manage the stressors and demands of caregiving. They are more likely to maintain a positive outlook, utilize effective coping strategies, and find meaning in their caregiving role. This, in turn, can lead to better mental and physical health outcomes for the informal caregiver, as well as improved care for the care recipient [35].

### 1.3. Literature Gap and the Present Study’s Aim

The COVID-19 pandemic was a specific contextual factor that acted as an additional stressor for informal caregivers, offering the opportunity to observe the interplay and dynamics among coping and emotional regulation strategies in these specific circumstances. Recent scientific evidence suggests that, during the COVID-19 pandemic, informal caregivers experienced reduced psychological well-being, including increased anxiety, depression, stress, caregiving-related distress, and burden [36,37]. Understanding how the psychological condition of informal caregivers was impacted and identifying the key mechanisms of this is crucial for developing targeted future interventions.

However, there is a noticeable gap in the current literature, as research on the psychological mechanisms driving these states—particularly regarding the interplay of the coping and emotional regulation strategies used by informal caregivers during the pandemic—is still limited.

Thus, this research study aimed to explore and gain insight into the complex patterns of relationships among emotional regulation, coping strategies, and resilience concerning symptoms of anxiety and depression in Italian informal caregivers by using psychometric network analysis.

Psychometric network analysis has the potential to provide valuable insights into the interconnections among emotional strategies, coping mechanisms, and resilience in informal caregivers during the COVID-19 pandemic, influencing the patterns of anxiety and depression symptoms. [38,39,40,41]. Network analysis allows for an examination of the mutual interrelations among each component of the network while controlling for the effects of other variables, thereby revealing the ‘true’ connections that persist after accounting for the influences of other nodes.

## 2. Materials and Methods

A cross-sectional study design was used. An online survey created in the software Qualtrics (version 2021) was used to collect data.

### 2.1. Participants

The inclusion criteria were being an informal caregiver; being above 18 years old; fluently speaking Italian; completing the assessment; and providing informed consent to participate in the study. The period of data collection was during the COVID-19 pandemic, from July to December 2021. Participants were recruited online, and the link to the survey was distributed via posts and advertisements on social media (e.g., Facebook). The snowball sampling method was used. In the presentation of the study, it was explained that the study aimed to address people who provided unpaid assistance to a loved one with a medical condition, including relatives and friends. Moreover, the definitions of informal and formal caregivers were provided. Then, in the survey, a specific question asked each participant if they were the informal caregiver of a person and more specific questions about caregiving. All participants were volunteers who received no economic remuneration for their participation in the study.

All participants provided informed consent. This project was approved by the Ethical Committee for the Psychological Research of the University of Padova (approval code number: 4722).

### 2.2. Measures

The following self-reporting assessment tools were administered to the participants.

*Socio-demographic sheet*: this included questions about age, biological sex, relationship status, and occupational status, but also about the caregiving role, such as the type of relationship with the patient and their medical condition.

*Emotion Regulation Questionnaire (ERQ).* The ERQ [25,26] is a 10-item self-report tool to measure emotional regulation strategies with two dimensions: cognitive reappraisal (6 items) and expressive suppression (4 items). The ERQ employs a 7-point Likert scale, ranging from 1 (= “strongly disagree”) to 7 (= “strongly agree”), with higher scores indicating greater use of the respective emotional regulation strategy. The questionnaire has demonstrated strong psychometric properties in both clinical and nonclinical populations [26]. This study utilized the Italian version of the ERQ, in which the internal consistency values were good both for reappraisal (Cronbach’s alpha = 0.84) and suppression (Cronbach’s alpha = 0.72) [25]. The internal consistency values in this study were good as well, both for cognitive reappraisal (Cronbach’s alpha = 0.87, omega = 0.93, greatest lower bound (glb) = 0.87) and expressive suppression (Cronbach’s alpha = 0.76, McDonald’s omega = 0.77, glb = 0.72).

*Coping Orientations to Problem Experienced-Nuova Versione Italiana-25 (COPE-NVI-25).* The COPE-NVI-25 [31] evaluates the coping strategies individuals can use when experiencing stressful events. The tool is composed of 25 items and assesses 5 different coping strategies through sub-scales: “Positive attitude” (6 items), “Problem orientation” (5 items), “Transcendent orientation” (4 items), “Social support” (5 items), and “Avoidance strategies” (5 items). Items are scored on a 6-point Likert-type scale from 1 (= “I do not do this at all”) to 6 (= “I usually do this a lot”), with higher scores indicating greater use of the strategy. In the original Italian validation [31], the Cronbach’s alpha values were good (avoidant strategies (alpha = 0.68), transcendent orientation (alpha = 0.95), positive attitude (alpha = 0.74), social support (alpha = 0.81), problem orientation (alpha = 0.77)). Also, in this study, the internal consistency values were good for all subscales—avoidant strategies (alpha = 0.71, omega = 0.72, glb = 0.69), transcendent orientation (alpha = 0.98; omega = 0.98, glb = 0.98), positive attitude (alpha = 0.90, omega = 0.91, glb = 0.94), social support (alpha = 0.90, omega = 0.90, glb = 0.92), and problem orientation (alpha = 0.86, omega = 0.86, glb = 0.85).

*Resilience Scale-14 items (RS-14).* The RS-14 [42] measures trait resilience with 14 items scored on a 7-point Likert-type scale from 1 (= “Strongly disagree”) to 7 (= “Strongly agree”). The total scores range from 14 to 98. Scores below 73 indicate very low (<56), low (57–64), or a resilience level on the low end (65–73), respectively, while scores above 74 indicate moderate (74–81), moderately high (82–90), or high (>91) resilience. An example of an item is: “I usually manage one way or another”. The internal consistency value in the Italian validation of the RS-14 was good (alpha = 0.88), as well as in this study (alpha = 0.91, omega = 0.93, glb = 0.92).

*Generalized Anxiety Disorder-7 items (GAD-7).* The GAD-7 [43,44] evaluates both cognitive and somatic symptoms of anxiety. It consists of 7 items scored on a 4-point Likert-type scale ranging from “not at all” (= 0) to “nearly every day” (= 3). The GAD-7 has a unidimensional factorial structure, the total score ranges from 0 to 21, and higher scores indicate more anxious symptoms. The internal consistency value was good in the original study (alpha = 0.92) [43], in the Italian validation (alpha = 0.92) [44], and in this study (alpha = 0.90, omega = 0.92, glb = 0.90).

*Patient Health Questionnaire-9 items (PHQ-9).* The PHQ-9 [44,45] evaluates symptoms of depression with 9 items scored on a 4-point Likert-type scale ranging from “not at all” (= 0) to “nearly every day” (= 3). The PHQ-9 has a unidimensional factorial structure and its total score ranges from 0 to 27, with higher scores indicating more severe levels of depression symptoms. The internal consistency value was good in the original validation (alpha = 0.86–0.89) [45], in the Italian validation (alpha = 0.92) [44], and in this study (alpha = 0.87, omega = 0.89, glb = 0.87).

### 2.3. Statistical Analysis

All statistical analyses were conducted with R software (version 4.3.3) [46] in the R Studio environment [47], using the packages bootnet [48], qgraph [49], networkTools [50], ggplot2 [51], and psych [52].

Preliminary analyses were conducted before performing the network analysis. First, the item informativeness level was evaluated within each scale [53]; an item is poorly informative when its standard deviation (SD) is 2.5 SDs below the average SD of all items [53,54]. Second, potential node redundancy was assessed; two nodes in a network are deemed redundant if they exhibit more than 75% statistically equal correlations with all other nodes in the network [50].

Regarding the main statistical analysis, a psychometric network analysis was used to disentangle the relationships among cross-sectional psychological variables [40,55,56]. The popular Pairwise Markov Random Field (PMRF) model was used to estimate psychological networks [57,58]. In a PMRF network, nodes represent psychological variables that are connected by undirected and weighted edges. Such edges show that these two variables are conditionally dependent given the other nodes in the network (i.e., one node can predict the other after controlling for the others). Absent edges indicate that these nodes are conditionally independent (i.e., one node cannot predict the other after controlling for the others). A PMRF applied to polychoric partial correlations on continuous data originates a Gaussian Graphical model (GGM) [57].

To control for spurious connections, the Graphical Least Absolute Shrinkage and Selection Operator (GLASSO) regularization algorithm was used to estimate more parsimonious network models [55,56,59,60], as it shrinks low correlations to 0, thus resulting in a sparse network where likely spurious connections are removed. The GLASSO uses a tuning/penalization parameter λ to control sparsity (a higher lambda and higher sparsity). Then, to retrieve the ‘true’ network structure, model selection was operated by minimizing the extended Bayesian information criterion (EBIC) [61] by using a γ hyperparameter set at 0.5 to obtain fewer connections and hardly spurious ones. GLASSO regularization combined with EBIC model selection has been demonstrated to be particularly effective in retrieving the true network structure [62,63], in particular when the true generating network is sparse (i.e., containing few edges), and EBIC-GLASSO has been shown to exhibit a high specificity overall (i.e., not estimating non-existing edges), but a variable sensitivity (i.e., accurately estimating the existing edges).

Then, a nonparametric bootstrap with 1000 resampling was used to evaluate the network stability and a Correlation Stability coefficient (CS coefficient), with values equal to or above 0.5 indicating the optimal stability [48,64]. Then, the strength centrality index of the nodes was estimated, expressing the number of edges connected with a node [65,66,67].

## 3. Results

### 3.1. Participants

The sample consisted of 351 informal caregivers (330 females, mean age 49.33 ± 11.104). Table 1 reports the socio-demographic descriptive statistics of the sample. Most of the participants were married (59.8%), had graduated high school (53.0%), and were employed (56.1%).

Most of the informal caregivers were assisting one of their parents (49.8%), children (22.2%), partner (17.9%), sibling (2.6%), or other (e.g., grandparent, relatives, or friends) (7.4%). The medical conditions of the patients assisted by the informal caregivers were dementia (13.11%), Parkinson’s disease (10.54%), Alzheimer’s disease (7.12%), neurological (11.11%), genetic conditions (9.97%), oncological conditions (4.84%) autism (4.84%), and cardiovascular conditions (1.14%), followed by others. The caregivers assisted their patients for a minimum of 3 months to a maximum of 45 years, with a mean duration of 9.06 years (SD = 7.94). Most of the caregivers (69.79%) lived with the patients they assisted, dedicating several hours per week to care. The hours of assistance ranged from 10 hours per week to 24 hours a day, 7 days a week, with a mean of 89 hours per week (SD = 67).

### 3.2. Preliminary Analysis

Detailed descriptive statistics of the variables included in the network are reported in Table 2, showing that the variables have low skewness and kurtosis values and are normally distributed.

**Figure 1:**
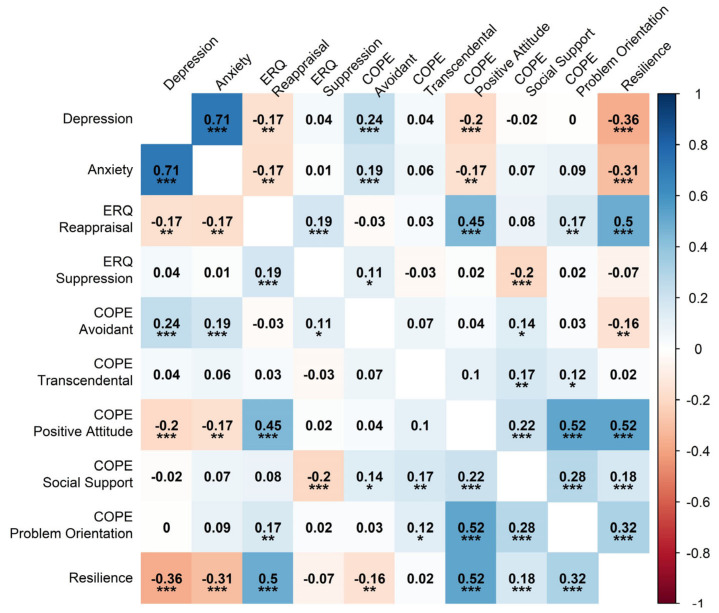
Heatmap of Pearson’s zero-order correlations between variables in the network. Note: ERQ = Emotion Regulation Questionnaire; COPE = Coping Orientations to Problem Experienced-Nuova Versione Italiana. * = *p <* 0.05, ** = *p <* 0.01, *** = *p <* 0.001.

Figure 1 depicts a heatmap of Pearson’s correlations between the psychological variables in the network, showing that none of the variables had a too-strong zero-order association. The highest correlation was between symptoms of depression and anxiety (r = 0.71). None of the items proved to be poorly informative (2.50 SD < mean), and no variable was potentially redundant with others in the network.

### 3.3. Psychometric Network Analysis

The network model retrieved by the EBIC-GLASSO algorithm was based on the network weights. The weights matrix of the network is reported in Table 3, with the majority being positive edge weights (in green) and only two negative ones (in red). The strongest positive edge was between anxiety symptoms and depression symptoms (0.647), followed by the relationships between coping through a positive attitude and coping through orientation to the problem (0.421), the edge between emotional regulation through reappraisal and resilience (0.340), between positive attitude and emotional regulation through reappraisal (0.244), between coping through problem orientation and anxiety symptoms (0.138), and finally, the smallest positive edge was between avoidant coping and depression symptoms (0.135). The strongest negative edge was between coping through social support and emotional regulation through suppression (−0.204), followed by the association between depression symptoms and resilience (−0.146).

The Supplementary Materials contain additional information about the edges’ stability across the bootstrap replications (Figure S1), the difference in the edges’ weights (Figure S2), and the differences in the nodes’ strengths (Figure S3).

**Figure 2:**
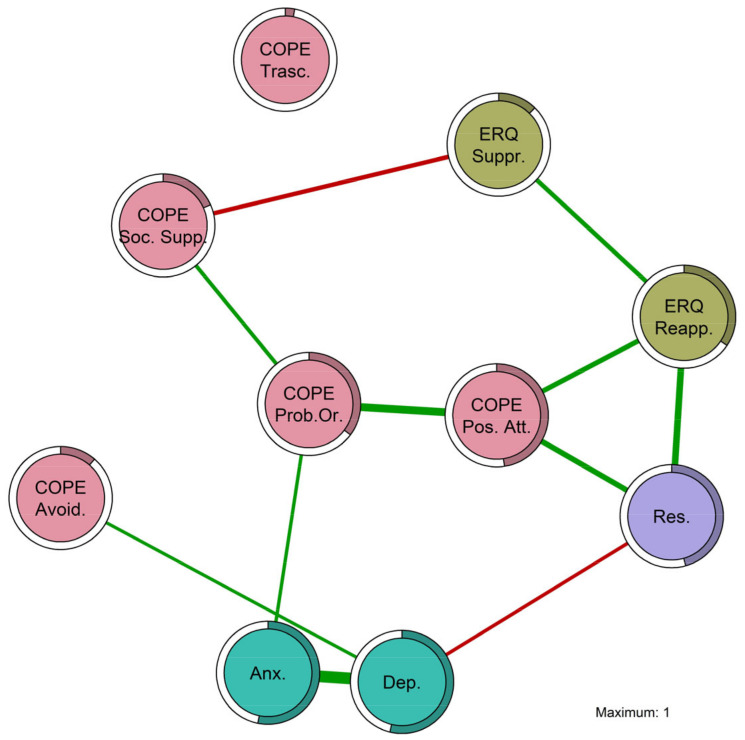
Plot of the EBIC-GLASSO network structure in informal caregivers. Note: ERQ = Emotion Regulation Questionnaire; COPE = Coping Orientations to Problem Experienced-Nuova Versione Italiana. ERQ Reapp. = ERQ Reappraisal; ERQ Suppr. = ERQ Suppression; COPE Avoid. = COPE Avoidant; COPE Trasc. = COPE Transcendental; COPE Pos. Att. = COPE Positive Attitude; COPE Soc. Supp. = COPE Social Support; COPE Prob. Or. = COPE Problem Orientation; Res. = Resilience; Anx. = anxiety symptoms; Dep. = depression symptoms.

Based on the network weights, it was possible to create the graph shown in Figure 2. Nodes from the same group have the same color: coping strategies are in red; emotion regulation strategies are in green; resilience is in violet; and negative psychological outcomes (anxiety and depression symptoms) are in light blue. In Figure 2, the circles around the nodes represent the amount of explained variance of that node by the other nodes, namely predictability. The last column of Table 3 reports the predictability values, showing that the most predictable variables were depression symptoms (0.538), anxiety symptoms (0.532), and coping through a positive attitude (0.476).

Regarding the network results and inference, both edge strengths and node centrality were evaluated. There were interesting relationships of conditional dependence between coping strategies, emotional regulation strategies, resilience, and negative psychological outcomes in informal caregivers. The edges in the network define the most likely parsimonious true network structure, explaining the multivariate data of the measured observed variables in informal caregivers. The estimated relationships between variables (edges) hold even when controlling for all the others in the network.

#### 3.3.1. Network Stability

A network stability analysis is crucial to verify that the retrieved model is robust against sampling variability and can be replicated in other samples. The non-parametric bootstrap with 1000 replications showed that this network model had optimal stability with a CS coefficient of 0.75 for edges, meaning that up to 75% of the participants could be dropped from the sample and the edges would still be stable. The CS coefficient for strength was 0.67. These optimal CS coefficients indicate that the network results are robust and reliable. Figure S5 in the Supplementary Materials shows a graph of the network stability metrics through case dropping.

#### 3.3.2. Centrality Indices

**Figure 3:**
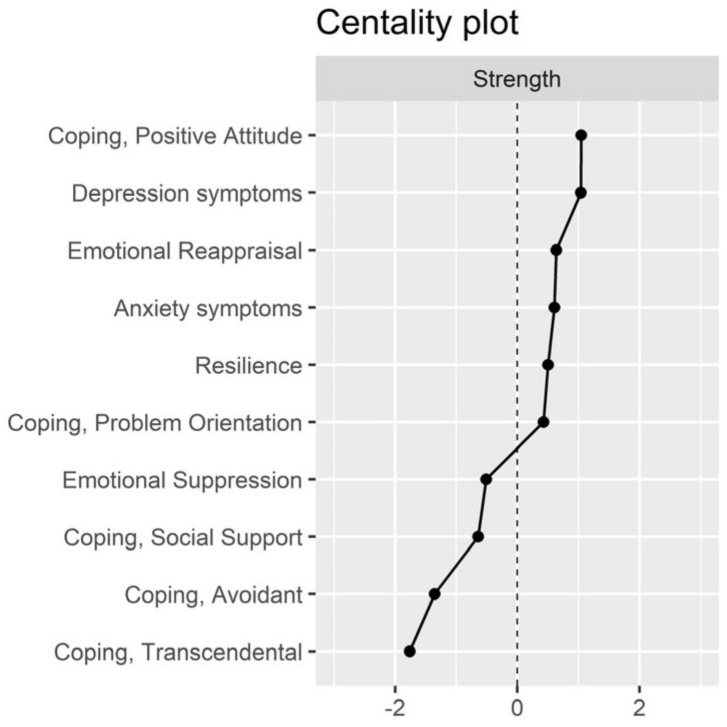
Plot of standardized centrality indices.

Figure 3 shows a plot of the standardized centrality indices. An analysis of the centrality indices showed that the nodes with the highest strength (higher numbers of relationships with other nodes) were coping through a positive attitude, depression symptoms, emotional regulation through reappraisal, anxiety symptoms, and resilience. The nodes with the lowest strength were transcendental coping, avoidant coping, coping through social support, and suppression of emotional expression.

Overall, the findings of the centrality indices indicated that coping through a positive attitude, depression symptoms, emotional reappraisal, anxiety symptoms, resilience, and coping oriented to the problem had a higher importance in the network of constructs. The other coping strategies (e.g., transcendental, avoidant, and social support) seemed to have a less important role, as well as the emotional regulation strategy of suppression.

## 4. Discussion

Recent research evidence indicates that informal caregivers faced reduced psychological well-being, including increased anxiety symptoms, depression symptoms, caregiving-related distress, and burden, in the context of the COVID-19 pandemic [36,37]. Understanding the pandemic’s impact on their mental health is crucial for developing effective interventions. However, research on the psychological mechanisms of informal carers during the COVID-19 pandemic, particularly their coping and emotion regulation strategies, is limited. Thus, this research sought to explore the complex relationships between emotional regulation, coping strategies, resilience, and symptoms of anxiety and depression among Italian informal caregivers using psychometric network analysis.

### 4.1. Interpretation of Research Findings

The psychometric network analysis allowed us to identify the most parsimonious model explaining the complex relationships among various psychological variables in a large sample of informal caregivers during the COVID-19 pandemic. Anxiety and depression symptoms were common negative psychological outcomes. To cope with the psychological impact of distressing conditions like health emergencies (e.g., COVID-19, severe acute respiratory syndrome—SARS, and Middle East respiratory syndrome—MERS) [68], informal caregivers often used coping strategies, emotional regulation strategies, and resilience. The network model revealed strong and stable associations that persisted even when accounting for the influence of all other variables in the network.

The network model identified a quadruplet of protective nodes that were the most important in the network: (i) coping through a positive attitude; (ii) emotional regulation through reappraisal; (iii) coping through problem orientation; and (iv) trait resilience. These adaptive constructs were positively connected and may reinforce each other, suggesting that individuals who use one strategy are likely to use the others as well. These constructs are crucial for supporting and promoting the psychological health of informal caregivers. Additionally, the scientific literature indicates that adaptive coping strategies contribute to resilient outcomes across various populations [69]. Given the correlational nature of the study, multiple pathways are possible. For example, trait resilience (e.g., believing in one’s ability to handle difficulties) can lead to the use of cognitive reappraisal as an emotional regulation strategy (e.g., changing one’s thinking) and adopting a positive attitude toward problems (e.g., accepting the situation and finding the silver lining). This positive attitude, in turn, is linked to a greater orientation to the problem to manage it (e.g., engaging in practical problem-solving).

In this specific network, coping through social support appeared to play a secondary rather than primary role in promoting positive health outcomes. Transcendental coping was isolated and not connected to other nodes, indicating that it was conditionally independent and may not be fundamental in this network. Avoidant coping was positively linked to depressive symptoms, which was expected since avoidance is a maladaptive strategy. While it may offer short-term relief, in the long term, avoidance often leads to procrastination, rumination, and eventually sadness and depression [70]. Conversely, higher depression symptoms may lead individuals to adopt more avoidance-based coping strategies to avoid additional negative feelings, ultimately reinforcing their depressive states. However, this approach can end up reinforcing the individual’s depressed and deactivated state [71].

Interestingly, coping through problem orientation was positively associated with anxiety symptoms. This may have been because, after accounting for depression symptoms, the remaining association reflected a common activation effect. Medium to moderate levels of anxiety can be useful in dealing with challenges [72], but excessive anxiety becomes maladaptive.

In line with the literature, trait resilience was strongly negatively associated with depression symptoms, indicating that informal caregivers with lower resilience are more likely to experience depressive feelings. Vice versa, informal caregivers with depressive symptoms may be less prone to rely on their resilience skills. Emotion regulation through cognitive reappraisal and suppression were positively associated, as both aim to regulate negative emotions, with reappraisal being considered functional, while suppression is maladaptive [25]. Suppression of emotional expression is negatively linked to coping via social support, likely because those who suppress emotions may be less willing to share them. Conversely, individuals who do not use social support—whether intentionally or due to circumstances such as isolation—may attempt to cope with unpleasant emotions alone, often resorting to suppression [73,74].

Importantly, coping and resilience are fundamental constructs that apply across various populations and contexts [69]. While this study focused on caregivers, the scientific literature shows that these constructs are also relevant to a broader range of individuals facing life’s challenges, stress, and adversity (i.e., healthcare professionals, students, and individuals experiencing trauma) [30,75,76,77]. However, across different populations, research indicates that adaptive coping strategies, such as problem-solving and seeking social support, are consistently linked to higher resilience [78]. From a broader perspective, these insights can inform the development of programs aimed at enhancing coping skills and fostering resilience across various settings, with beneficial results for different populations [79].

### 4.2. Implications for Clinical and Research Fields

Regarding the clinical implications of this research, constructs with higher centrality indices played a key role in the network and may be potential targets for clinical interventions focused on strengthening coping and emotional regulation strategies to mitigate the adverse psychological effects of a distressing pandemic on informal caregivers and their patients. Regarding research implications, psychometric network analysis proved to be a valuable methodology for understanding the complex relationships among various psychological variables and disentangling their unique associations.

Understanding the factors that enhance the psychological health and resilience of informal caregivers is crucial for developing services and treatments that bolster their coping strategies, resilience, and mental health [80,81]. Moreover, including trait resilience in illness and psychopathology models emphasizes individual strengths despite risk factors, potentially improving health, well-being, and quality of life in at-risk contexts (for a review, [82]). Understanding and promoting resilience in informal caregivers is, therefore, vital in clinical psychology, supporting the well-being of both informal caregivers and care recipients [83].

### 4.3. Limitations, Strengths, and Future Research

This study is not free of limitations. First, the cross-sectional design prevented establishing directed causal relationships among the variables. Longitudinal experimental studies are needed to observe symptom trajectories over time and differentiate between and within effects on medium- to long-term outcomes [84,85,86,87]. Second, the network edges were undirected, so causality in either direction was plausible. Third, due to the pandemic, only online self-report assessments were used, which are prone to biases, but were necessary to reach a broad sample of informal caregivers. Fourth, the sample had a moderate heterogeneity in terms of socio-demographic characteristics, which needs further exploration in future studies. Finally, the sample had fewer males, reflecting the demographics of informal caregivers, where more females, with a greater age range, take on informal caregiving roles—probably because of social and cultural reasons. Importantly, biological sex may influence coping strategies and resilience, as suggested by the recent literature on sex and gender differences [88,89].

Some strengths of this research are noteworthy. First, this is one of the few studies focusing on the psychological health of informal caregivers in the Italian context. Second, the measures used in this study were all well-validated tools with good psychometric properties, which represents a fundamental prerequisite for the validity and replicability of the study results [90]. Third, a large sample of informal caregivers with various socio-demographic characteristics representative of the population was used. Fourth, another strength lies in the ecological validity of the present research, as it provided an ecological sample from a clinical population of informal caregivers who, despite having some heterogeneity, were all living in the same conditions during the pandemic, representing a life-disrupting event [91]. Fifth, the large sample size allowed for the use of a modern and advanced psychometric approach as network analysis to gain insights into the dynamics of psychological constructs. Sixth, the network model proved to be robust and stable despite sampling variability, with optimal indices supporting the replicability of these results in future studies.

Regarding the generalizability and extensibility of the results, it is important to emphasize that the findings of this study can be applied to various contexts beyond the COVID-19 pandemic. Although the COVID-19 pandemic exacerbated clinical phenomena such as anxiety and depression in caregivers, these results could also be extensible and replicable in other contexts.

Future research will test if these results can be replicated even in the absence of global health crises like the COVID-19 pandemic, considering that psychological mechanisms might change depending on circumstances. Studies should aim to replicate these findings in larger samples and extend them beyond Italy through cross-cultural comparisons with northern and eastern countries where social, cultural, economic, and welfare systems differ. Future studies can also test the roles of age and sex as moderators of the relationship between coping strategies and resilience in informal caregivers, with a particular focus on the less-studied male informal caregivers. Biological sex might moderate the relationships among constructs, such as males using less social support and more problem-oriented coping [88,89]. Future research should investigate how the duration and severity of patients’ conditions could significantly affect the caregiver burden, influencing resilience, coping strategies, emotional regulation, anxiety, and depression. This would provide a more nuanced understanding of how patients’ illness severity and duration impact caregivers’ psychological outcomes and help to tailor interventions to caregivers’ specific needs at different stages of their caregiving journey. Additionally, research should simultaneously consider the psychological conditions of both informal caregivers and their patients, who also experience high levels of distress, to target their intertwined relationships by using dyadic interventions [92,93]. Moreover, greater attention should be given to the social, cultural, and environmental factors in which individuals live, adopting an ecological perspective [80,94].

## 5. Conclusions

This research pinpointed the key constructs involved in informal caregiving and suggested relevant targets for interventions [95,96]. Coping strategies like maintaining a positive attitude may significantly mitigate the adverse emotional effects of pandemics, particularly depression and anxiety symptoms. Targeting these constructs could decrease overall network activation, alleviating psychological issues and promoting emotional well-being in informal caregivers. Network analysis is a robust and effective methodology for highlighting the constructs linked to emotional well-being, identifying intervention targets, and exploring the relationships among constructs.

Given the growing number of informal caregivers worldwide and the health challenges they face, caregiving is being increasingly recognized as a public health issue that needs institutional attention to improve the health of a significant population segment [4,94]. 

## Figures and Tables

**Table 1:** Descriptive statistics of the sample variables.

	Females (N = 330)	Males (N = 21)	Total (N = 351)
Age, mean (SD)	49.221 (11.182)	50.952 (9.882)	49.325 (11.104)
Range	18–85	38–68	18–85
Relationship status			
Single	44 (13.3%)	4 (19.0%)	48 (13.7%)
Engaged	28 (8.5%)	1 (4.8%)	29 (8.3%)
Cohabiting	36 (10.9%)	3 (14.3%)	39 (11.1%)
Married	197 (59.7%)	13 (61.9%)	210 (59.8%)
Separated	10 (3.0%)	0 (0.0%)	10 (2.8%)
Divorced	9 (2.7%)	0 (0.0%)	9 (2.6%)
Widow	6 (1.8%)	0 (0.0%)	6 (1.7%)
Education			
Primary school	4 (1.2%)	0 (0.0%)	4 (1.1%)
Middle school	42 (12.7%)	4 (19.0%)	46 (13.1%)
High school	175 (53.0%)	11 (52.4%)	186 (53.0%)
Bachelor’s degree	31 (9.4%)	1 (4.8%)	32 (9.1%)
Master’s degree	48 (14.5%)	4 (19.0%)	52 (14.8%)
Post-graduate	29 (8.8%)	1 (4.8%)	30 (8.5%)
None	1 (0.3%)	0 (0.0%)	1 (0.3%)
Occupational status			
Student	7 (2.1%)	0 (0.0%)	7 (2.0%)
Working student	8 (2.4%)	0 (0.0%)	8 (2.3%)
Employed	183 (55.5%)	14 (66.7%)	197 (56.1%)
Unemployed	64 (19.4%)	3 (14.3%)	67 (19.1%)
Retired	43 (13.0%)	1 (4.8%)	44 (12.5%)
Other	25 (7.6%)	3 (14.3%)	28 (8.0%)

**Table 2:** Descriptive statistics of psychological variables in the network.

	Mean	Sd	Median	Min	Max	Skewness	Kurtosis	Se	ω
PHQ-9	13.031	6.012	12	0	27	0.401	−0.484	0.321	0.89
GAD-7	12.422	4.924	12	0	21	0.109	−0.941	0.263	0.92
ERQ Reappraisal	26.04	8.101	26	6	42	−0.2	−0.285	0.432	0.93
ERQ Suppression	15.148	5.709	15	4	28	−0.082	−0.676	0.305	0.77
COPE Avoidance	9.513	3.653	9	5	29	1.282	2.133	0.195	0.72
COPE Transcendental	9.538	6.483	8	4	24	1.089	−0.181	0.346	0.98
COPE Positive Attitude	21.897	7.65	21	6	36	0.142	−0.723	0.408	0.92
COPE Social Support	14.513	6.423	13	5	30	0.67	−0.309	0.343	0.90
COPE Problem orientation	19.402	5.729	19	5	30	0.144	−0.799	0.306	0.86
Resilience	60.943	16.02	62	20	98	−0.029	−0.645	0.855	0.93

Note: PHQ-9 = Patient Health Questionnaire 9 items; GAD-7 = Generalized Anxiety Disorder 7 items; ERQ = Emotion Regulation Questionnaire; COPE = Coping Orientations to Problem Experienced-Nuova Versione Italiana; α = Cronbach’s alpha; ω = McDonald’s omega.

**Table 3:** Undirected weights matrix of the EBIC-GLASSO network model and nodes’ predictability. Red highlights negative weights, while green highlights positive ones. Higher predictability is indicated by a more intense green.

	Depression Symptoms	Anxiety Symptoms	ERQ Reappraisal	ERQ Suppression	COPE Avoidant	COPE Transcendental	COPE Positive Attitude	COPE Social Support	COPE Problem Orientation	Resilience		Predictability
Depression symptoms	-	0.647	0	0	0.135	0	0	0	0	−0.146		0.538
Anxiety symptoms	0.647	-	0	0	0	0	0	0	0.138	0		0.532
ERQ Reappraisal	0	0	-	0.211	0	0	0.244	0	0	0.34		0.344
ERQ Suppression	0	0	0.211	-	0	0	0	−0.204	0	0		0.123
COPE Avoidant	0.135	0	0	0	-	0	0	0	0	0		0.114
COPE Transcendental	0	0	0	0	0	-	0	0	0	0		0.029
COPE Positive Attitude	0	0	0.244	0	0	0	-	0	0.421	0.265		0.476
COPE Social Support	0	0	0	−0.204	0	0	0	-	0.167	0		0.184
COPE Problem Orientation	0	0.138	0	0	0	0	0.421	0.167	-	0		0.352
Resilience	−0.146	0	0.34	0	0	0	0.265	0	0	-		0.456

Note: ERQ = Emotion Regulation Questionnaire; COPE = Coping Orientations to Problem Experienced-Nuova Versione Italiana.

## Data Availability

The data presented in this study are available on request from the corresponding author due to privacy reasons.

## Supplementary Materials

The following supporting information can be downloaded at: https://www.mdpi.com/article/10.3390/bs14080709/s1, Figure S1: Stability of edge weights across bootstrap replications; Figure S2: Differences in edge weights across bootstrap replications; Figure S3: Differences in Strength across bootstrap replications; Figure S4: Correlation Stability index across bootstrap replications.
