# Supplementary material for: Emotional Regulation, Coping, and Resilience in Informal Caregivers: A Network Analysis Approach
Source: Behav Sci (Basel). 2024 Aug 13;14(8):709. doi: 10.3390/bs14080709 (PMC11351438; doi:10.3390/bs14080709)

Supplementary materials

Emotional Regulation, Coping, and Resilience in Informal Caregivers: A Network Analysis Approach

Anna Panzeri, Gioia Bottesi, Marta Ghisi, Cecilia Scalavicci, Andrea Spoto, and Giulio Vidotto

Department of General Psychology, University of Padua, Italy

Figure S1. Stability of edge weights across bootstrap replications

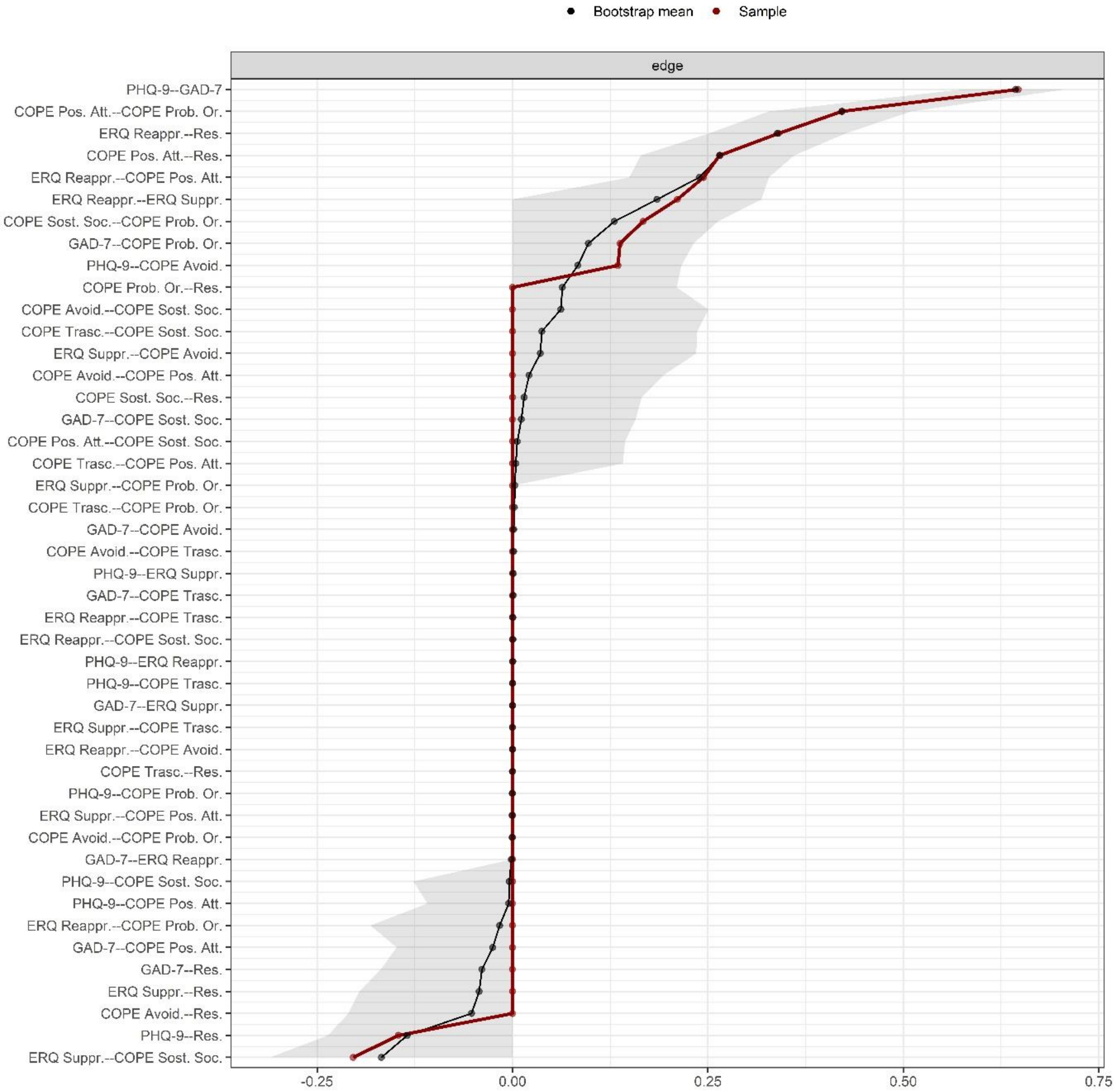

Figure S2 Differences in edge weights across bootstrap replications

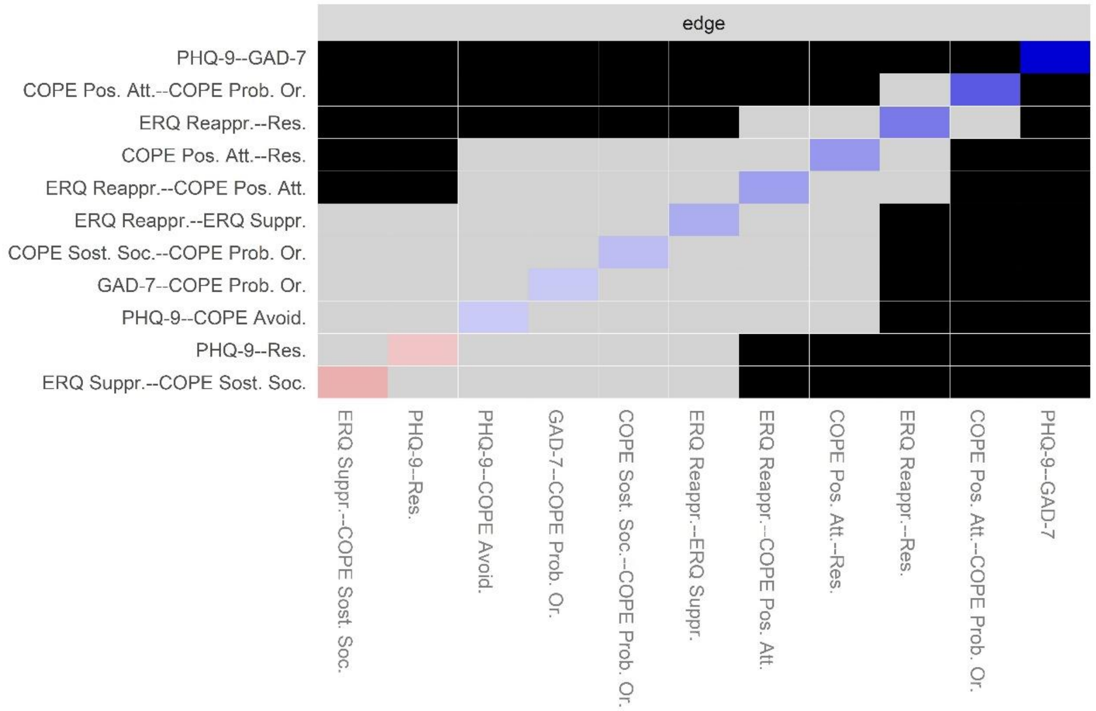

Figure S3. Differences in Strength across bootstrap replications

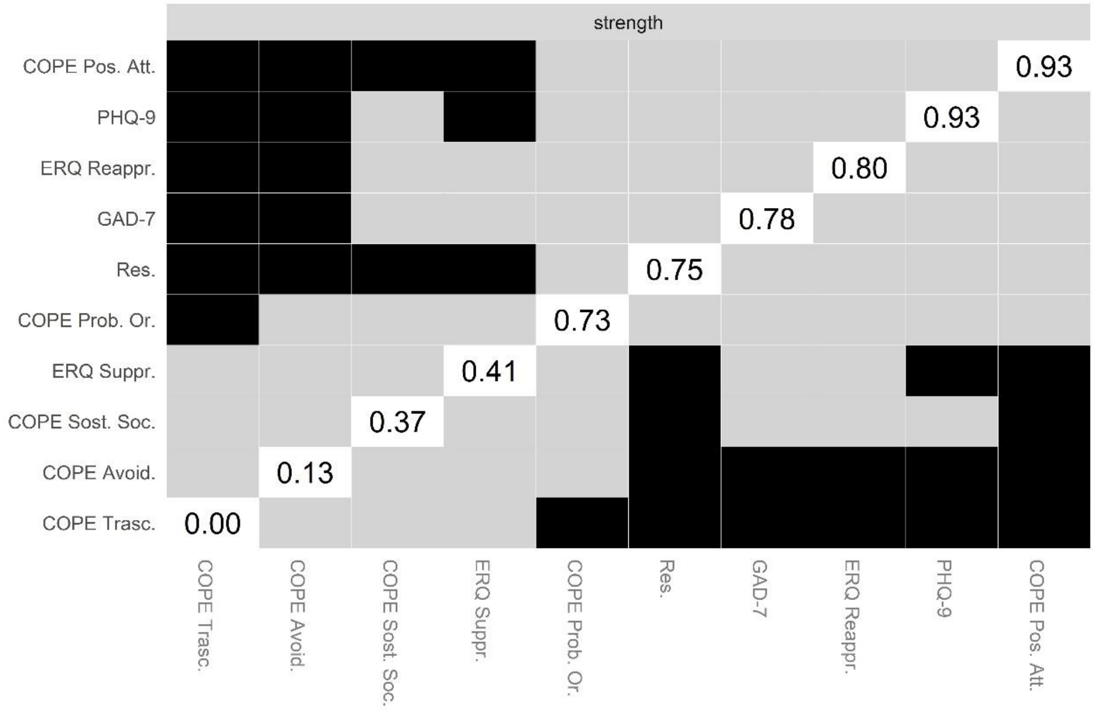

Figure S4. Correlation Stability index across bootstrap replications

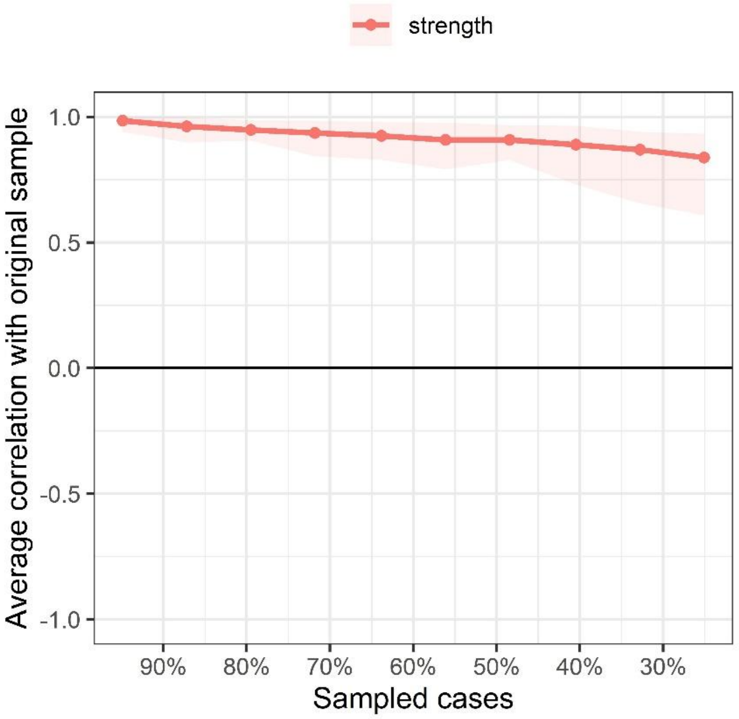

Supplement: Supplementary file 1 [file behavsci-14-00709-s001.zip › behavsci-3108654-supplementary.pdf]
